# Immunosignature Screening for Multiple Cancer Subtypes Based on Expression Rule

**DOI:** 10.3389/fbioe.2019.00370

**Published:** 2019-11-29

**Authors:** Lei Chen, XiaoYong Pan, Tao Zeng, Yu-Hang Zhang, YunHua Zhang, Tao Huang, Yu-Dong Cai

**Affiliations:** ^1^School of Life Sciences, Shanghai University, Shanghai, China; ^2^College of Information Engineering, Shanghai Maritime University, Shanghai, China; ^3^Shanghai Key Laboratory of Pure Mathematics and Mathematical Practice (PMMP), East China Normal University, Shanghai, China; ^4^Key Laboratory of System Control and Information Processing, Ministry of Education of China, Institute of Image Processing and Pattern Recognition, Shanghai Jiao Tong University, Shanghai, China; ^5^IDLab, Department for Electronics and Information Systems, Ghent University, Ghent, Belgium; ^6^Key Laboratory of Systems Biology, Institute of Biochemistry and Cell Biology, Chinese Academy of Sciences, Shanghai, China; ^7^Shanghai Institute of Nutrition and Health, Shanghai Institutes for Biological Sciences, Chinese Academy of Sciences, Shanghai, China; ^8^Anhui Province Key Laboratory of Farmland Ecological Conservation and Pollution Prevention, School of Resources and Environment, Anhui Agricultural University, Hefei, China

**Keywords:** cancer subtype, expression rule, immunosignature, multi-class classification, feature selection

## Abstract

Liquid biopsy (i.e., fluid biopsy) involves a series of clinical examination approaches. Monitoring of cancer immunological status by the “immunosignature” of patients presents a novel method for tumor-associated liquid biopsy. The major work content and the core technological difficulties for the monitoring of cancer immunosignature are the recognition of cancer-related immune-activating antigens by high-throughput screening approaches. Currently, one key task of immunosignature-based liquid biopsy is the qualitative and quantitative identification of typical tumor-specific antigens. In this study, we reused two sets of peptide microarray data that detected the expression level of potential antigenic peptides derived from tumor tissues to avoid the detection differences induced by chip platforms. Several machine learning algorithms were applied on these two sets. First, the Monte Carlo Feature Selection (MCFS) method was used to analyze features in two sets. A feature list was obtained according to the MCFS results on each set. Second, incremental feature selection method incorporating one classification algorithm (support vector machine or random forest) followed to extract optimal features and construct optimal classifiers. On the other hand, the repeated incremental pruning to produce error reduction, a rule learning algorithm, was applied on key features yielded by the MCFS method to extract quantitative rules for accurate cancer immune monitoring and pathologic diagnosis. Finally, obtained key features and quantitative rules were extensively analyzed.

## Introduction

Liquid biopsy (i.e., fluid biopsy) involves a series of clinical examination approaches, including sampling and analysis, on non-solid suspected pathogenic tissues, such as blood (Crowley et al., 2013), amniotic fluid (Ilas et al., 2000), and cerebrospinal fluid (Hiemcke-Jiwa et al., 2018). At present, liquid biopsy is applied in three main fields: cancer studies (Condello et al., 2018; Mithraprabhu and Spencer, 2018), heart attack diagnosis (Ogawa et al., 1983), and prenatal diagnosis (Sun et al., 2015). For heart attack diagnosis, the circulating endothelial cells are usually the inspected targets, reflecting the extent of damage on the integrity and permeability of heart, and related vessels (Ogawa et al., 1983). As for prenatal diagnosis, cell-free fetal DNA reflects the genomic characteristics of the infant, applicable for the development of monitoring, and diagnosis of genetic disorders (Sun et al., 2015). In cancer studies, liquid biopsy has been used for the identification of cancer biomarkers to monitor the progression of tumorigenesis and predict the prognosis. In 2014, a specific study (Stafford et al., 2014) on the evaluation of immune status of cancer presented a novel method for tumor-associated liquid biopsy, i.e., monitoring of cancer immunological status by the “immunosignature” of patients.

Immunosignature describes a typical reductionist biomarker paradigm assay that contributes to the representation of patients' immune responses but not the direct cancer status (Reiman et al., 2007; Stafford et al., 2014). Similar to traditional liquid biopsy on the basis of tumor-associated biomarkers, the identification of immunosignature in clinical examinations aims at the evaluation of the pathogenic conditions of cancer patients and the prediction of personalized cancer prognosis. However, such approach focuses on the immune elimination capacity on tumor cells of each patient so as to provide an auxiliary diagnosis rather than the direct tumor progression, invasion, and metastasis conditions. Thus, the major work content and the core technological difficulties for the monitoring of cancer immunosignature would be the recognition of cancer-related immune-activating antigens by random sequence peptide microarray screening (Reiman et al., 2007). Peptides in such microarray that can be bound by patient peripheral blood-derived antibodies share the same epitopes as endogenous antigens, which are probably derived from tumor tissues (Stafford et al., 2014). Such high-throughput screening approaches are efficient and accurate to identify tumor-associated antigens.

According to Stafford et al. (2014), patients with different tumor subtypes have different antigen spectrum responses to the peripheral isolated antibodies, validating that immunosignature may be a novel monitoring parameter for cancer liquid biopsy. However, the wide clinical application of immunosignature-based cancer liquid biopsy has three major obstacles. First, the identified potential antigens of each patient are outnumbered. Thus, the tumor-derived antigens, even the specific immune evaluation biomarkers, are hard to identify. Second, screening the whole randomized antigen assay of all the potential antibodies for each patient is impractical because of expensive and time-consuming burden. Third, the qualitative recognition and analysis of antigens are not accurate and efficient enough for personalized cancer monitoring, which requires quantitative standards to be established. Therefore, one key task of immunosignature-based liquid biopsy is the identification of shared cancer immune evaluation biomarkers together with their absolute quantity ranges. For instance, the identification of typical tumor specific antigens should be in a qualitative and quantitative manner.

To solve such problem from clinics, in this study, we reused the peptide microarray data that detected the expression level of potential antigenic peptides derived from the tumor tissues. To remove the detection differences induced by chip platforms, we independently analyzed the potential antigen distribution data from two datasets obtained from different chip platforms. Several machine learning algorithms were used in this study. The Monte Carlo Feature Selection (MCFS) (Draminski et al., 2008) was adopted to evaluate the importance of features in two datasets, respectively, resulting in a feature list. The incremental feature selection (IFS) (Liu and Setiono, 1998) was applied on the feature list to extract optimal features and build an optimal classifier based on a given classification algorithm. In addition, the repeated incremental pruning to produce error reduction (RIPPER) algorithm (Cohen, 1995) was performed on essential features that were produced by the MCFS method to construct quantitative classification rules. Altogether, we not only identified the common distributed cancer-associated antigen patterns but also established a series of quantitative rules for accurate cancer immune monitoring and pathologic diagnosis. Obtained patterns and rules were analyzed in the end of this paper.

## Methods and Materials

### Datasets

We downloaded the peptide microarray data from Gene Expression Omnibus under Accession Number GSE52582 (Stafford et al., 2014). It included two datasets. Dataset-1 (from GSE52580) was measured with 10K immunosignaturing peptide microarray version 2 and included 240 samples from six groups (Brain cancer, Breast cancer, Esophageal cancer, Multiple myeloma, Pancreatic cancer, and Healthy control). Each group had 40 samples. Dataset-2 (from GSE52581) was measured with ASU_random-sequence peptide microarray and included 1,516 samples from 15 groups of various diseases. Additional information of the samples can be found in Stafford et al. (2014). Dataset-1 contained 9,786 peptides, whereas dataset-2 contained 10,371. However, dataset-2 had missing values. To infer the missing values, we adopted the K-Nearest Neighbor (K = 10) method from R package.

### Feature Selection

The purpose of feature selection is to distinguish important features from unimportant ones in datasets for a certain machine learning task. In this study, we used MCFS (Draminski et al., 2008) to capture key genes (features) for classifying samples from different diseases and to determine interpretable rules. We obtained the optimal genes with strong distinctions between different types of diseases through IFS method (Liu and Setiono, 1998).

#### Monte Carlo Feature Selection

In this study, MCFS (Draminski et al., 2008) was applied to select important genes. MCFS is a random sampling multivariate feature selection method based on original features. Assuming there are *M* original features, we randomly select some feature subsets, each of which includes randomly selected *m* features (*m*<<*M*) in original *M* ones. Then, multiple decision trees are generated and evaluated in the bootstrapping datasets from the original dataset, where the number of generated decision trees is *p*. After repeating the above process *t* times, *t* feature subsets and *p* × *t* decision trees are obtained.

The relative importance (RI) provides a score of each feature for its performance in the above decision trees. The RI score of a feature *g* is calculated by

$$\begin{array}{l} RI_{g}=\overset{p\times t}{\underset{\tau =1}{\sum }}{\left(wAcc\right)}^{u}IG\left(n_{g}\left(\tau \right)\right){\left(\frac{no.in\;n_{g}\left(\tau \right)}{no.in\;\tau }\right)}^{v}, \end{array}$$

where *wAcc* is the weighted accuracy, and *n*_*g*_(τ) is a node of feature *g* in decision tree τ. The information gain of *n*_*g*_(τ) is expressed as *IG*(*n*_*g*_(τ)), and *no*.*in n*_*g*_(τ) is the number of training samples in *n*_*g*_(τ), where *u* and *v* are different weighting factors with a default value of 1.

#### Rule Learning

In this study, we adopt the MCFS method to analyze two peptide microarray data from GEO. Each feature is assigned a RI value. Some informative features are further extracted by the MCFS method with a permutation test on class labels and one-sided student's *t*-test. These informative features are used to construct interpretable rules, which can clearly display the classification procedures for a given sample. In detail, these features are first reduced by Johnson Reducer algorithm (Johnson, 1974; Ohrn, 1999), such that remaining features have similar classification ability to all informative features. Then, remaining features are fed into the RIPPER algorithm (Cohen, 1995), which is a set-based rule learning algorithm, to determine simple, and interpretable rules for classifying samples from different disease types. The procedures of RIPPER algorithm for constructing rules can be found in our one previous study (Wang et al., 2018). Each rule describes the relationship between conditions and outcomes. Here, the rules are expressed as IF-THEN relationships based on detailed expression values. For example, IF Peptide 1<=0.7 AND Peptide 2>=1.02, THEN type = “Brain cancer.”

#### Incremental Feature Selection

IFS (Liu and Setiono, 1998) is an ideal feature ranking method with a supervised classifier. It filters the input and result in a set of optimal features for distinguishing different sample sets/classes with the best performance. Features in the feature list are ranked in descending order according to their RI values, and IFS is performed on such feature list. The combination of high-ranked features should help the classification model perform well because high-ranked features are important for classification. Here, we perform IFS in two steps.

First, we constructed a series of feature subsets with a large step size 10 to create feature subsets with high performance. In feature subsets *F* = [$F_{1}^{1},F_{2}^{1},\ldots ,F_{m}^{1}$], the i-th feature subset contains 10 × *i* features, that is, $F_{i}^{1}=\left[f_{1},f_{2},\ldots ,f_{i\times 10}\right]$. A classifier with a certain prediction engine is built to evaluate samples composed of each feature subset by 10-fold cross-validation (Kohavi, 1995). After all feature subsets have been tested, we can obtain a feature interval [min, max], which helps the classifier provide a good prediction performance. Based on the interval [min, max] from the first stage, a series of feature subsets $\left[F_{\min+1}^{2},F_{\min+2}^{2},\ldots ,F_{\max}^{2}\right]$ is built to further accurately extract the optimal features. The final optimal feature subset with the optimal performance can be obtained finally. The classifier with such optimal feature subset is called optimal classifier.

### Random Forest

A random forest is a meta-classifier that contains a large number of tree classifiers (Breiman, 2001). For classification, its output categories are determined by aggregating votes from different decision trees. The main idea of building a random forest, which is widely used in computational biology, is to ensemble a large number of decision trees (Pan et al., 2010; Zhao et al., 2018; Zhao R. et al., 2019; Zhao X. et al., 2019). Some differences always exist between each decision tree and other decision trees in the decision tree set. To avoid over-fitting, the random forest averages the prediction results of all decision trees to reduce the prediction variance. Although causing a small increase in bias and some loss of interpretability, the ensemble model usually has improved performance.

### Support Vector Machine

Support vector machine (SVM) (Cortes and Vapnik, 1995) is a supervised learning algorithm based on statistical learning theory and is suitable for dealing with many biological problems (Pan and Shen, 2009; Mirza et al., 2015; Chen et al., 2017b, 2018a; Cai et al., 2018; Cui and Chen, 2019; Zhou et al., 2019). It can build models for linear and non-linear classification problems. The SVM model represents the samples as points in data space such that the samples of the individual categories can be separated after data mapping, and then the categories can be determined based on which side of the interval samples fall. The basic principle is to infer the hyperplane with the maximum margin between two types/classes of samples. In addition, SVM can be extended to multi-class problems based on its basic binary-class problem. For multi-class problems, SVM generally adopts the strategy of “One vs. the Rest.” In this study, we use the sequence minimum optimization algorithm (Platt, 1998), which is widely adopted for SVM learning.

### Performance Measurement

This study employed the Matthew's correlation coefficient (MCC) (Matthews, 1975; Gorodkin, 2004) as the key measurement for evaluating the performance of classifiers because it is deemed as a balanced measurement even if sizes of classes are of great differences. The original MCC was proposed by Matthews (1975), which was designed for binary classification and has wide applications (Chen et al., 2017a, 2018b; Li et al., 2019). Here, two datasets (Dataset-1 and Dataset-2) contain more than two classes. Thus, the multi-class version of MCC was used, which was proposed by Gorodkin (2004). To calculate such MCC, two matrices are first constructed, say *X* and *Y*, where *X* is a 0-1 matrix representing the predicted class of each sample and *Y* is also a 0-1 matrix indicating the true classes of all samples. Then, such MCC is defined as

$$\begin{array}{l} MCC=\frac{\mathrm{cov}\left(X,Y\right)}{\sqrt{\mathrm{cov}\left(X,X\right)\mathrm{cov}\left(Y,Y\right)}}, \end{array}$$

where cov(·, ·) stands for the covariance of two matrices. To date, such MCC has been applied to evaluate performance of different multi-class classifiers (Salari et al., 2014; Schmuker et al., 2014; Zhang et al., 2019). For convenience, such MCC is also called MCC in the following text.

## Results

**Figure 1:**
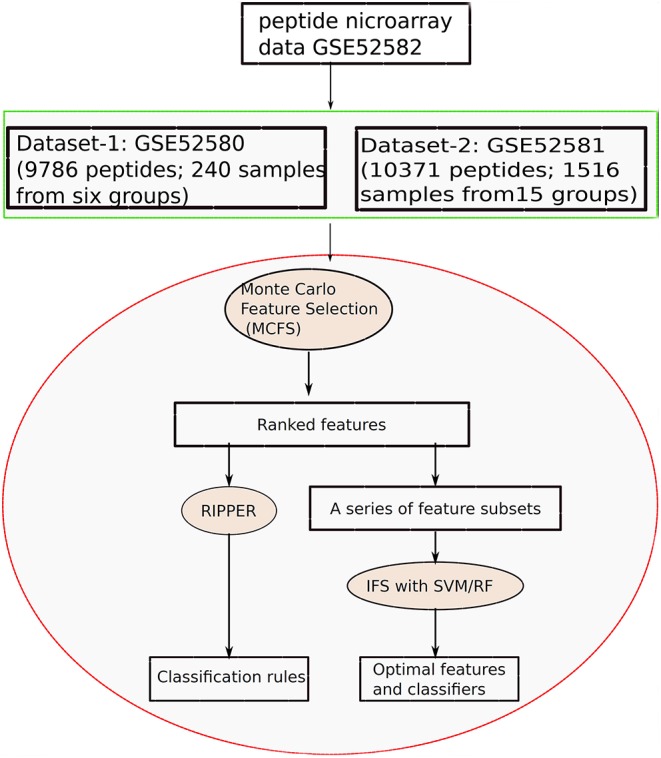
Entire procedures to investigate the peptide microarray data from Gene Expression Omnibus with advanced machine learning algorithms.

In this study, we applied machine learning methods to classify samples from different types of diseases, which mainly cover two datasets. One consists of five cancers and heath control (called Dataset-1), and the other one consists of 15 diseases (called Dataset-2). We use the same computational pipeline for analyzing these two datasets separately. Entire procedures are shown in Figure 1.

### Results on Dataset-1

**Figure 2:**
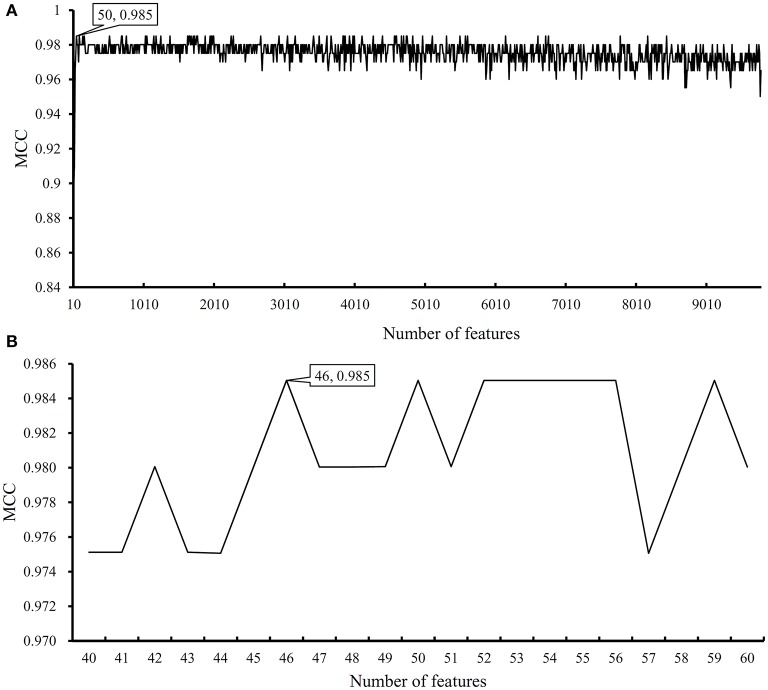
Performance of random forest (RF) on different feature subsets. **(A)** Performance of RF on feature subsets with step 10; **(B)** Performance of RF on feature subsets with top 40–60 features.

**Figure 3:**
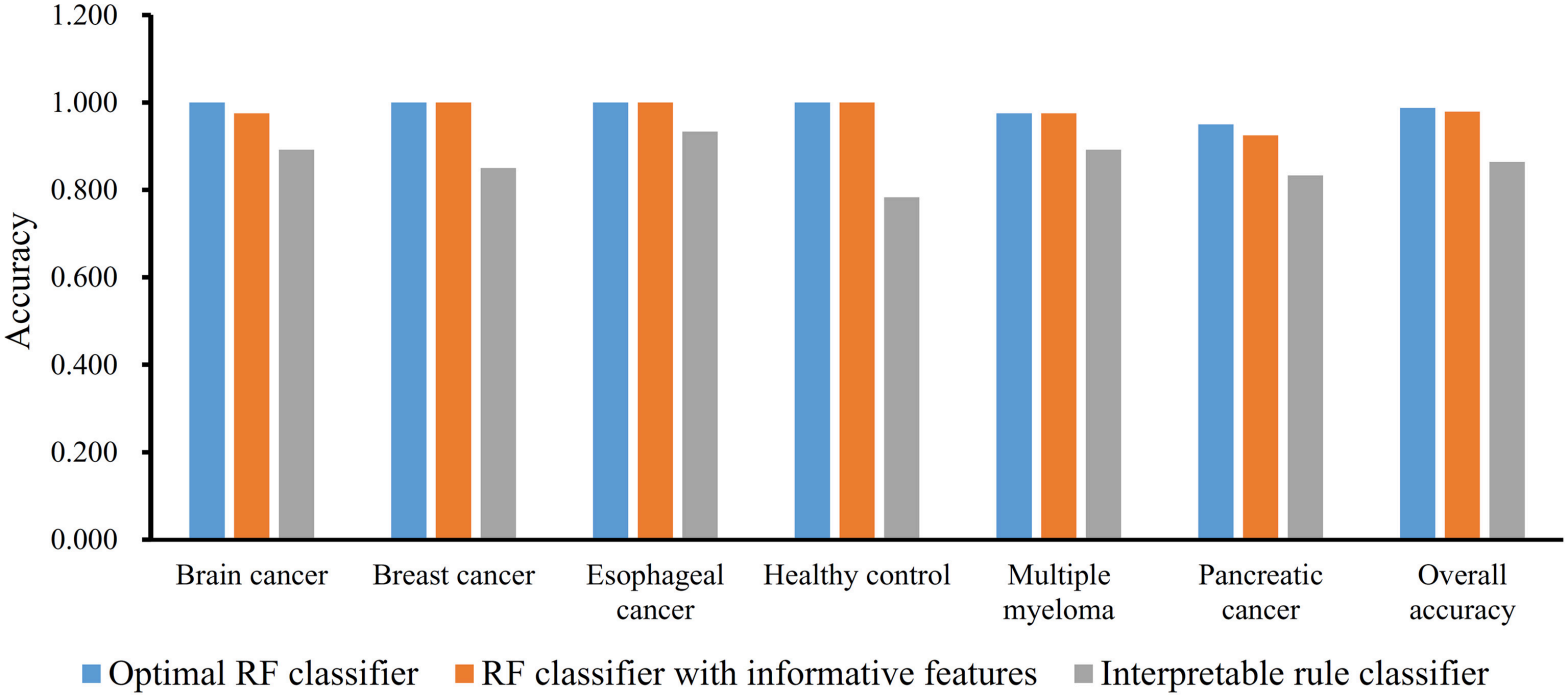
Performance of three classifiers on six groups in dataset-1 and overall accuracy.

We first run the feature selection method to detect potential antigenic peptides associated with six classes. The RI scores for all peptides are given in Table S1. In general, using all available features may not yield the best performance. To identify the optimum number of features with the best performance for classifying samples from the six classes in this dataset, we run the IFS with an integrated RF classifier. We first run the RF on the series of feature subsets with a step 10. The performance of RF corresponding to different numbers of features is given in Table S2. For an easy observation, the MCCs on different feature subsets is illustrated in Figure 2A, from which we can see that the highest MCC is obtained when top 50 features are used. Thus, we determine an interval range [40, 60] for further investigation. Then, on a series of feature subsets with a step 1 between the range [40, 60]. The performance of RF on these feature subsets is shown in Figure 2B. We obtain the best MCC value of 0.985 when top 46 features are used (Table 1). These 46 features are deemed to be optimal features for RF and a RF classifier with these features are called optimal RF classifier. The detailed performance, including accuracies on six classes and overall accuracy, is illustrated in Figure 3. Such classifier gives perfect performance on four classes and overall accuracy is 0.988, suggesting the high performance of the optimal RF classifier. Of note, we also have an in-house assessment that RF can outperform SVM on Dataset-1.

**Table 1:** The classification performance on two datasets.

**Dataset**	**Classifier**	**Optimum number of features**	**MCC**
Dataset 1	RF	46	0.985
Dataset 2	SVM	2,846	0.952

The MCFS method can output some informative features for any input dataset. For Dataset-1, 517 informative features are extracted, which are the first 517 features in Table S1. It is interesting to investigate the performance of RF on these features. By 10-fold cross-validation, we obtain the MCC of 0.975, which is lower than that yielded by above-mentioned optimal RF classifier. Its detailed performance is displayed in Figure 3. The overall accuracy is 0.979. On individual accuracies of six classes, none of them can exceed corresponding accuracy yielded by the optimal RF classifier. Thus, the IFS method can actual find out the optimal feature subspace for RF, thereby providing higher performance.

**Figure 4:**
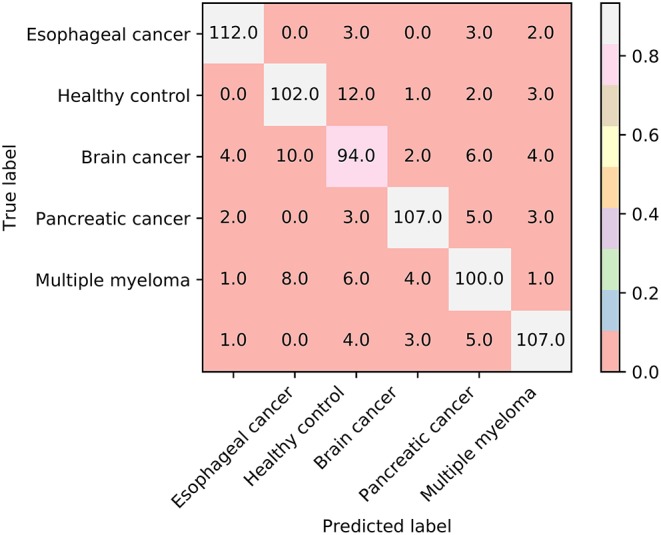
The confusion map of seven classification rules on dataset-1.

Furthermore, we also employ the Johnson Reducer and RIPPER algorithms to construct interpretable rules based on 517 informative features. To test the performance of rules yielded by these two algorithms, 10-fold cross-validation is performed thrice. We yield the MCC of 0.837. The confusion map (Figure 4) shows the misclassification among six classes. Accordingly, the accuracies on six classes are counted and illustrated in Figure 3. They are all much lower than those of the optimal RF classifier. Although the rule classifier provided much lower performance, they can provide a clear classification procedure and indicate the differences between different classes, thereby giving more biology insights. Accordingly, we further applied Johnson Reducer and RIPPER algorithms on all samples in Dataset-1, obtaining seven classification rules, which are listed in Table 2.

**Table 2:** Seven detected rules for classifying different diseases in dataset-1.

**Rules**	**Criteria**	**Subtype**
Rule1	CSGAGFEGTGLRCSLLCLDR <= 0.795	Esophageal cancer
Rule2	CSGFQPMRYPFQDPYHGYGW <= 1.056 CSGADFVTYATRRVQFMMHK <= 1.611	Pancreatic cancer
Rule3	CSGFLMEHQNLLERSEDAKA <= 0.569 CSGGEGIQATYHKVGGNFLG >= 1.238	Healthy control
Rule4	CSGTYEPHLVYLATFTDGIP <= 0.870	Healthy control
Rule5	CSGEKIGMEQHYNQWIELMR >= 1.036	Multiple myeloma
Rule6	CSGADFVTYATRRVQFMMHK >= 1.282	Brain cancer
Rule7	Others	Breast cancer

### Results on Dataset-2

**Figure 5:**
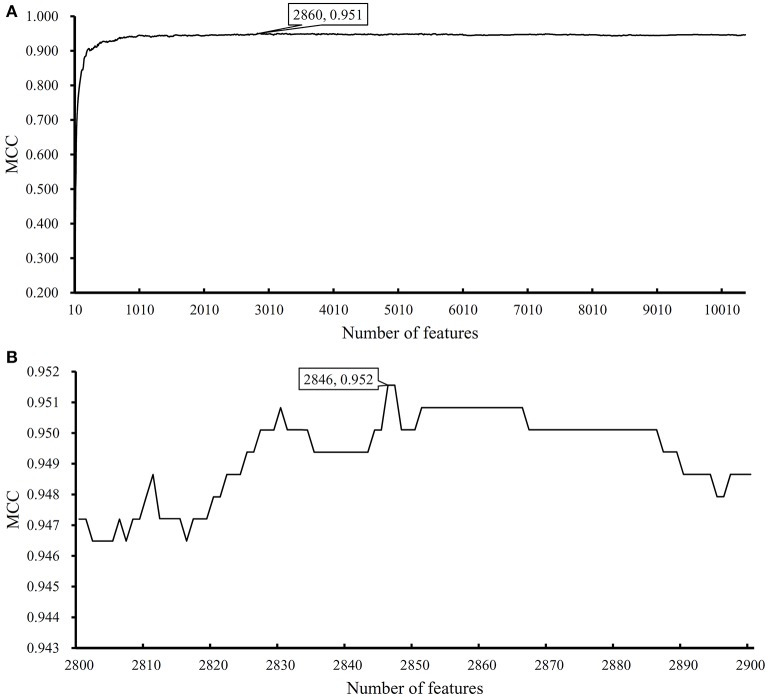
Performance of support vector machine (SVM) on different feature subsets. **(A)** Performance of SVM on feature subsets with step 10; **(B)** Performance of SVM on feature subsets with top 2,800–2,900 features.

**Figure 6:**
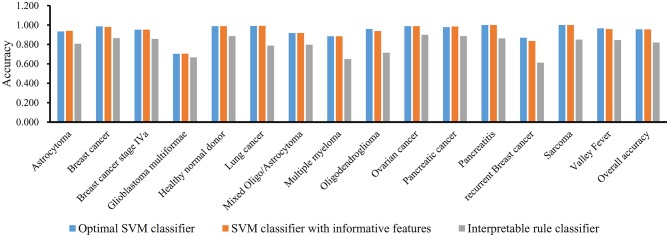
Performance of three classifiers on 15 groups in dataset-2 and overall accuracy.

We performed the same analysis as above on Dataset-2. We first use MCFS to rank the input features, whose RI scores are given in Table S3. Then, we run IFS with an integrated SVM on the samples consisting of features from the generated feature subsets with a step 10. The performance of SVM corresponding to different numbers of features is provided in Table S4. Figure 5A shows the performance of SVM, evaluated by MCC, on above-constructed feature subsets. The highest MCC is 0.951 when top 2,860 features are adopted. Then, we obtain an interval [2,800, 2,900] for further investigation. To further extract the optimum number of features, we run the SVM on the samples consisting of the features from a series of feature subsets generated from the interval with a step 1. The performance of SVM on these feature subsets is shown in Figure 5B. We obtain the best MCC value of 0.952 when the top 2,846 features are used (Table 1). Thus, these top 2,846 features are termed as optimal features for SVM and the SVM classifier with these features are called optimal SVM classifier. The individual accuracies on 15 classes and overall accuracy are illustrated in Figure 6. The overall accuracy is 0.956, two classes receive the highest accuracy of 1.000, other 10 classes receive the accuracy higher than 0.900. All these suggest the high performance of the optimal SVM classifier. Of note, we also have an in-house assessment that SVM can outperform RF on Dataset-2.

Similar to Dataset-1, the MCFS method extracts 3,264 informative features. With these 3,264 features, an SVM classifier on Dataset-2 is built and evaluated by 10-fold cross-validation. the MCC is 0.949, which is slightly lower than that of the optimal SVM classifier. Figure 6 shows the accuracies on 15 groups and overall accuracy. The overall accuracy is 0.954. For 15 individual accuracies, such classifier generated higher accuracies on two classes than the optimal SVM classifier, while on four classes, it yields lower accuracies. Altogether, the optimal SVM classifier gives higher performance.

**Figure 7:**
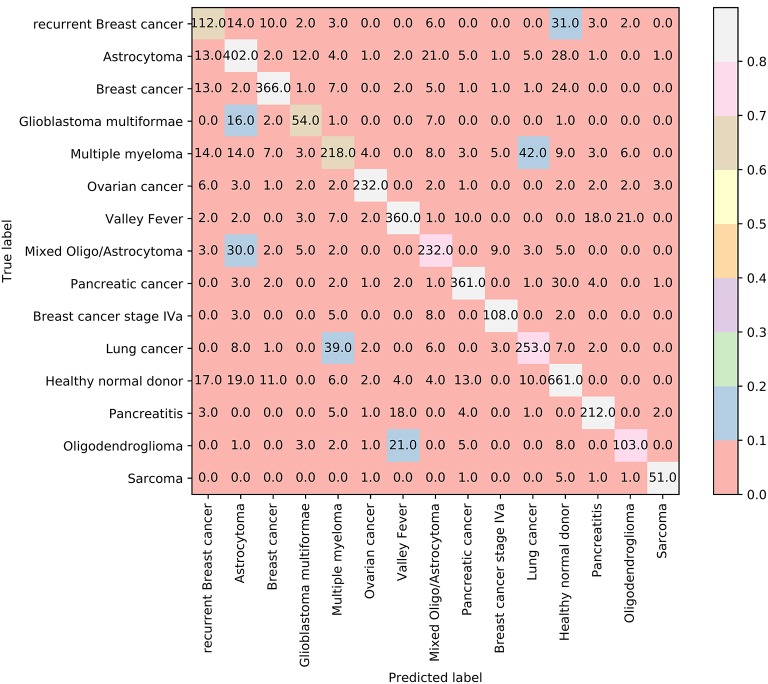
The confusion map of 42 classification rules on dataset-2.

In addition, we also adopt Johnson Reducer and RIPPER algorithms to construct classification rules based on 3,264 informative features. Ten-fold cross-validation is used to evaluate the performance of rules yielded by these two algorithms and such procedures are executed thrice, producing the MCC of 0.801, which is much lower than that of the optimal SVM classifier. The corresponding confusion map is shown in Figure 7. The accuracies of 15 classes and overall accuracy are illustrated in Figure 6. The rule classifier yields lower accuracies on all 15 classes compared with those of optimal SVM classifier. Likewise, 42 classification rules are constructed by applying Johnson Reducer and RIPPER algorithms on all samples in Dataset-2, which are listed in Table 3.

**Table 3:** Forty-two detected rules for classifying different diseases in dataset-2.

**Rules**	**Criteria**	**Subtype**
Rule1	HQKNDSANTVITTWLTRGSC>=5.265	Sarcoma
Rule2	MNVHYAAQDVINFGAHQGSC>=1.497 RENQHEIGVALARSHKMGSC<=0.427	Glioblastoma multiformae
Rule3	ELIAFRDFNWRGGVVAGGSC>=2.837 KWKQDYINNHFVKVNRTGSC>=1.622	Glioblastoma multiformae
Rule4	VWGKGGMYEAHYRRNGEGSC>=2.360 DEPKQYASWYTHWTNWAGSC>=3.931	Glioblastoma multiformae
Rule5	HDWNVAWELRRWKALIYGSC>=1.791 GTQPMVAWKDVYGIVVYGSC>=1.510 AAVAKRIAEQHMWMQVGGSC>=0.683	Breast cancer stage IVa
Rule6	KFPNEFRYRYNWRMQNPGSC>=7.729 AAVPKYINAMWKGYAPDGSC<=0.609	Breast cancer stage IVa
Rule7	FHWNMYKNSESLFEEKQGSC>=2.110	Oligodendroglioma
Rule8	PGLTHNTLQYMATVLSVGSC>=1.876 AAKFRTQWMHWMIWHHTGSC>=0.752	Oligodendroglioma
Rule9	PGLTHNTLQYMATVLSVGSC>=1.876 AAKFRTQWMHWMIWHHTGSC>=0.752	Oligodendroglioma
Rule10	QVNKAVSWYLVWHLWHQGSC>=1.183 AGLLWQWKGWDYIHEWNGSC<=0.466 LWFGTMPWHSIRAHDVHGSC<=0.616	Recurrent breast cancer
Rule11	HYNRYMVIIGNWGKQPIGSC<=0.509 GNSVRAFITVLMQIFFTGSC>=1.727 MKPLISYGPAWFGPLLWGSC>=0.538	Recurrent breast cancer
Rule12	GDQHQLEPPYKKNQYMIGSC>=1.857 RTGAGHTWDSTGHIQKVGSC>=0.968	Recurrent breast cancer
Rule13	RQNTIRSRQKINLGGGDGSC>=1.853 AADTGGFDLIWNEVKGHGSC>=1.130	Recurrent breast cancer
Rule14	PVGEVSSDYNRGPWRGTGSC>=1.977	Recurrent breast cancer
Rule15	SWIHGWLTITIYGFKERGSC>=1.631 AAVAKRIAEQHMWMQVGGSC<=0.331	Recurrent breast cancer
Rule16	DLVMPTNHESLSQLTGDGSC>=1.004 PFPNYPIYPMWMMHEREGSC>=2.888	Pancreatitis
Rule17	LERGHRADMAYRDTFPMGSC>=2.128 DQYELTQDLHVVKSYFAGSC<=0.512	Pancreatitis
Rule18	IKSRTGAEEIQIQMLLRGSC>=2.858	Pancreatitis
Rule19	LSERWAMGAHRDTASQTGSC>=1.540 ADDHEQWTEKMYKNQNMGSC>=0.523	Ovarian cancer
Rule20	ADVKMLWEWNDVKVLIIGSC>=4.318	Ovarian cancer
Rule21	VNFESFREPTFGSDGYSGSC>=2.353 EWYYDPRGGTGSFYMRTGSC>=0.972	Mixed Oligo/Astrocytoma
Rule22	LIVFTKGHRMYNDIPTNGSC<=0.434 APYTPQFFEAQTWWINGGSC>=1.146	Mixed Oligo/Astrocytoma
Rule23	YLSTSMEQEQEQVHGNWGSC>=2.247 ILDRRETAWNEHFSKFRGSC>=1.236	Mixed Oligo/Astrocytoma
Rule24	TVKKMYNGGLASKNALYGSC<=0.171 GHAVQGGLKRAHRVYKQGSC>=1.766	Mixed Oligo/Astrocytoma
Rule25	TQGVAHFGQTHYPYQLEGSC>=1.942 PHEEYMRQFHSAGQPTFGSC>=1.416 HHAFFNGEYMKMMSLSIGSC>=0.051	Lung cancer
Rule26	YVQEHAQWKNMWELANGGSC>=2.325 AADTGGFDLIWNEVKGHGSC<=0.806	Lung cancer
Rule27	FLKFMQKMSTVHIIWLNGSC<=0.118 ANQTHYDPTSSDMVWPKGSC>=1.071	Lung cancer
Rule28	TAKWYGIRNSQDEKVEAGSC>=1.756 AAKFRTQWMHWMIWHHTGSC>=0.750	Lung cancer
Rule29	YINSYPIAKPHGEEMQMGSC<=0.461 ETDKTINVREAAAHGMKGSC<=0.390	Multiple myeloma
Rule30	ERIYRDHFIHEHKANIIGSC<=0.545 NLFRWLWNRRHVWDQDRGSC>=1.092	Multiple myeloma
Rule31	TAHGKARDFDPAKNRYLGSC<=0.398	Multiple myeloma
Rule32	HFGIVISVMNEKEGALRGSC>=7.715	Multiple myeloma
Rule33	YFMWPFWWYSHVWGRDWGSC>=1.001 IITIWLDGGLMHDFEKPGSC>=1.028 AEMGFTSPERDQGASQEGSC<=1.493	Pancreatic cancer
Rule34	WWWFHSLGLLAHIKIALGSC>=1.122 FGFDFGDLWIIPDAIAMGSC>=1.068	Pancreatic cancer
Rule35	IISNTTMAVLWMLQSSRGSC>=1.429 ANQTHYDPTSSDMVWPKGSC>=0.758	Pancreatic cancer
Rule36	TYQRRMGGVRGQQPYNKGSC>=2.089 DGDPTAITNWWWETGNWGSC<=0.728	Breast cancer
Rule37	PKQHGRQQNQGIFKPMLGSC>=2.538 AGGNHLAIAFNAIFLNMGSC<=0.717	Breast cancer
Rule38	FKETAMPVLNYPVGVNEGSC>=1.959	Healthy normal donor
Rule39	GEASDNYKWWWDHVVYPGSC>=1.854	Astrocytoma
Rule40	FFYKKDFTPRHTFQNRRGSC<=0.529 AEMGFTSPERDQGASQEGSC<=0.586	Astrocytoma
Rule41	APMKNIVSAKTKDFAYMGSC<=0.324	Astrocytoma
Rule42	Others	Healthy normal donor

## Discussion

As described above, we screened and identified the core potential antigens that can be recognized by the free antibodies in the peripheral blood of patients with different diseases. All the identified peptides have been further mapped to their respective original proteins and corresponding encoding genes. Tumor-specific or tumor subtype-specific biomarkers shall be derived from tumor-associated genes or variations. Recent publications have confirmed that all the genes, which such peptides can be mapped to, are functionally related to tumorigenesis, validating the efficacy and accuracy of our prediction. Further, based on the abundance of each identified peptide, we set up a quantitative recognition standard for the accurate identification, accomplishing the quantitative analysis. The detailed analysis on each identified peptide and settled up rules is provided below.

### Immunosignature-Associated Genes

As mentioned in Section Results, some key features were extracted for each dataset. Their related genes are extracted and analyzed in this section.

#### Immunosignature-Associated Genes Derived From Dataset-1

In the first dataset, we screened and identified the candidate immunosignature antigens for six groups of samples: brain cancer, breast cancer, esophageal cancer, multiple myeloma, pancreatic cancer, and healthy controls.

The first identified peptide in dataset-1 is CSGHPFWHMKHESIYHIYYT, aligning to be a part of proteins (Altschul et al., 1990; Mount, 2007; Pruitt et al., 2014) prickle-like protein 1 and prickle-like protein 2 (Altschul et al., 1997). Encoded by functional genes PRICKLE1 and PRICKLE2, such two proteins participate in the regulation of the Wnt/beta-catenin signaling pathway (Daulat et al., 2012; Mermejo et al., 2014). As for its differential expression pattern in multiple tumor subtypes, the two proteins have been identified in multiple tumor subtypes, including brain cancer (Katoh and Katoh, 2003), breast cancer (Jaeger and Delacretaz, 1953), esophageal cancer (Shimo et al., 2004), and pancreatic cancer (Katoh and Katoh, 2003), but are rarely detected in multiple myeloma and normal controls, validating the distinctive capacity of such PRICKLE1- or PRICKLE2-derived antigen on distinguishing different tumor subtypes.

The second peptide CSGSAIKVMIEIFVMHPYIK can also be aligned to multiple reference proteins, such as protein orai-2 isoform b, angiopoietin-2 isoform a precursor, and zinc finger protein 462 isoform 2 (Altschul et al., 1990; Mount, 2007; Pruitt et al., 2014), indicating that such peptide may have multiple releasing sources (Altschul et al., 1997). The three mentioned sources of our identified peptides have all been confirmed to contribute to the clustering and recognition of each effective disease subtype. Take angiopoietin-2 isoform a precursor as an example. As a precursor of effective angiopoietin-2, such protein is a potential biomarker in brain cancer (Seifert et al., 2015), breast cancer (Han et al., 2016), multiple myeloma (Nowicki et al., 2017), and pancreatic cancer (Chou et al., 2016), but not esophageal cancer and normal controls, together with ANGPT2. Therefore, such protein-derived peptide may also contribute to the detailed distinction of multiple cancer subtypes.

The third identified peptide CSGTMNSEFQNTTRHVYIMS can be aligned to alstrom syndrome protein 1 (Altschul et al., 1990; Mount, 2007; Pruitt et al., 2014) with individual amino acid mismatches induced by tumor-derived genomic instability (Altschul et al., 1997). Encoded by gene ALMS1, such peptide loading protein has been only identified in multiple myeloma but not in other tumor subtypes and normal controls (Rajasagi et al., 2014; Braune et al., 2017). Therefore, such peptide may also be potential biomarkers for immunosignature recognition-based cancer diagnosis and prognosis in real-time because of its potential relationship with ALMS1, distinguishing unique cancer subtypes.

The following peptide CSGKSPRFHKGGIQYKVDWY can also be traced back to two effective tumor-associated proteins, namely, E3 ubiquitin-protein ligase NHLRC1 and gamma-tubulin complex component6, which participate in tumorigenesis (Altschul et al., 1997; Orlic et al., 2006; Martin et al., 2014) and contribute to the distinction of different tumor subtypes in our candidate tumor subgroups (Orlic et al., 2006; Martin et al., 2014). Therefore, based on dataset-1, all the identified qualitative immunosignature-associated peptides can be traced back to cancer immune antigens, validating the efficacy and accuracy of our prediction.

#### Immunosignature-Associated Genes Derived From Dataset-2

Apart from such analyzed optimal peptides identified on the first platform, we also tried to identify the core distinctive peptides derived from other platforms by the same computational approach, hoping to represent the comprehensive distinction capacity of immunosignature. In such dataset of samples (dataset-2), we screened out the candidate immunosignature antigens for 15 types of diseases, also including the normal controls.

The first identified peptide is FKETAMPVLNYPVGVNEGSC, aligning to three effective proteins succinate-semialdehyde dehydrogenase, mitochondrial isoform 1; succinate-semialdehyde dehydrogenase, mitochondrial isoform 2; and exosome complex component MTR3 (Altschul et al., 1990; Mount, 2007; Pruitt et al., 2014). These proteins have differential expression patterns in 15 sample subgroups. Considering the length limitation, we chose exosome complex component MTR3 as an example for detailed discussion. Mediating mRNA degradation (Houseley et al., 2007; Sandler et al., 2013), such protein participates in the pathogenesis of some disease subtypes, including some candidate subgroups such as breast cancer (Rosedale and Fu, 2010), but not other subtypes such as astrocytoma, glioblastoma multiforme, and lung cancer. Therefore, with specific expression pattern on proteomic level, the identified antigen may be differentially expressed in distinct diseases subtypes, validating the efficacy, and accuracy of our prediction.

The second identified peptide is SESTLAKIGVLGNLYDIGSC, derived from caspase-8, glutamate receptor ionotropic, and Kv channel interacting protein. Three proteins have been functionally connected to tumorigenesis. Taking Kv channel-interacting proteins (KCNIPs) as an example, members of the KCNIP family contribute to the inactivation of A-type potassium channels (Pruunsild and Timmusk, 2005; Moreau et al., 2016). Comparing with our candidate diseases list, such peptide can distinguish neural system-associated diseases from others because of the specific regulatory role of the KCNIP family in the nervous system (Néant et al., 2015; Moreau et al., 2016), validating the efficacy and accuracy of our approach.

The third identified peptide has a specific sequence of AQNADELEEYSASKHDGGSC, which can be realigned to multiple tumor-associated proteins, such as mediator of RNA polymerase II transcription subunit 1, protein FAM45A, and protein SETSIP (Altschul et al., 1990; Mount, 2007; Pruitt et al., 2014). All three proteins have differential expression patterns on the proteomic level in our 15 candidate disease subtypes (including health control). As a chromatin binding protein SETSIP, such gene participates in somatic cell reprogramming and cell differentiation (Margariti et al., 2012). Such protein is not expressed nor functioning in multiple tissue subtypes. Such identified protein only acts as a reprogramming regulator in vascular fibroblasts (Margariti et al., 2012) and human gastric epithelial cells (Fazeli et al., 2017). This finding reflects its specific tissue-restricted expression pattern and confirms that such protein is effective in distinguishing candidate 15 diseases by its specific tissue-restricted expression pattern.

As analyzed above, all identified peptides are derived from disease-associated genes/proteins, reflecting the abnormal expression pattern of certain genes/proteins under certain pathogenic conditions. Due to the limitation of the article length, all optimal peptides cannot be analyzed one by one. Peptides such as PMDEGFAQIAHQALINAGSC and VNHKPLLSGHSGVEWPSGSC also present their distinctive capacity for the candidate disease subgroups, validating the efficacy and accuracy of our prediction. Therefore, from one sight of qualitative analysis, immunosignature-based cancer liquid biopsy may be effective.

### Immunosignature-Associated Rules

In addition to the above analysis, we also applied two groups of quantitative analysis, recognizing a group of effective rules for the detailed distinction of each disease subtype. Due to the limitation of page length, we only focus the top-ranked three optimal rules of each datasets for following detailed data mining and discussion.

#### Immunosignature-Associated Rules From Dataset-1

The first rule of dataset-1 contributes to the recognition of samples derived from esophageal cancer by only one quantitative parameter, the low expression level of peptide CSGAGFEGTGLRCSLLCLDR. Recent publications have reported that such peptide is aligned to a group of specific proteins named phosphoinositide 3-kinase regulatory subunit 5 isoform 1/2 and sortilin-related receptor preproprotein (Altschul et al., 1990; Mount, 2007; Pruitt et al., 2014). Not all such identified proteins are lowly expressed in esophageal cancer, except for protein phosphoinositide 3-kinase regulatory subunit 5 (Zhang et al., 2018), validating the efficacy, and accuracy of our prediction. On the basis of the detailed expression level of such protein in serum provided by the Proteomics Database (Wilhelm et al., 2014; Schmidt et al., 2018) and the Cancer Proteomic Database (Arntzen et al., 2015), such protein has relatively high expression patterns in multiple tissue subtypes. As for its expression level in the serum of esophageal cancer patients, recent publications (Zhu et al., 2015; Peng et al., 2017) have also confirmed its low expression pattern, validating the prediction tendency of our quantitative rules.

As for the second rule, candidate peptides CSGFQPMRYPFQDPYHGYGW and CSGADFVTYATRRVQFMMHK derived from uracil-DNA glycosylase isoform UNG2, T-cell surface glycoprotein CD5 isoform 2, and transmembrane protein 33, N-acetylgalactosamine kinase isoform X10 contribute to the identification of pancreatic cancer. We chose T-cell surface glycoprotein CD5 isoform 2 and transmembrane protein 33 as two major peptide sources for detailed quantitative discussion. According to recent publications (Chu et al., 2003; Wörmann et al., 2014; Lu et al., 2015), these two proteins are lowly expressed in pancreatic cancer. Considering the detailed expression level in the Proteomics Database (Wilhelm et al., 2014; Schmidt et al., 2018) and the Cancer Proteomic Database (Arntzen et al., 2015), we further validated the low expression patterns of these genes.

As for the third quantitative rules, parameters CSGFLMEHQNLLERSEDAKA and CSGGEGIQATYHKVGGNFLG have been selected for the identification of healthy controls. By realigning to the Refseq protein database (Altschul et al., 1990; Mount, 2007; Pruitt et al., 2014), the two peptides have been confirmed to be derived from proteins 6-phosphofructo-2-kinase/fructose-2,6-bisphosphatase 3 (Clem et al., 2013) and zinc finger protein 296 (Fischedick et al., 2012), respectively. These identified proteins participate in specific pathogenesis with abnormal expression patterns (Fischedick et al., 2012; Clem et al., 2013). On the basis of the Proteomics Database (Wilhelm et al., 2014; Schmidt et al., 2018), the expression level of such two genes in blood is corresponding with our predicted threshold, validating our method's efficacy and accuracy.

#### Immunosignature-Associated Rules From Dataset-2

The first rule of our identified quantitative rule based on dataset-2 involves a unique peptide, HQKNDSANTVITTWLTRGSC, which can be further realigned to the protein interleukin-1 receptor type 2 with acceptable mismatches that contribute to the identification of sarcoma (Altschul et al., 1990; Mount, 2007; Pruitt et al., 2014). Different from other cancer subtypes, the overexpression of our identified peptide-derived protein interleukin-1 receptor type 2 promotes the initiation and progression of sarcoma (Boddul et al., 2014; Liu et al., 2015). As for the expression parameter we screened, such detailed expression level has also been confirmed based on the Cancer Proteomic Database (Arntzen et al., 2015).

In the second rule, MNVHYAAQDVINFGAHQGSC and RENQHEIGVALARSHKMGSC have been picked up as quantitative parameters for the identification of samples from glioblastoma multiforme patients. Re-aligning (Altschul et al., 1990; Mount, 2007; Pruitt et al., 2014) to effective proteins hydrocephalus-inducing protein and laminin subunit gamma-3 precursor, such rule corresponds to recent publications and related databases. On the basis of our quantitative rules, hydrocephalus-inducing protein has a relatively high expression level (>1.47) and laminin subunit gamma-3 precursor has a relatively low expression level (<0.42) at the proteomic level. Such two expression tendencies have already been confirmed by recent publications (Peles et al., 2004; Lathia et al., 2012). Considering that few reports focused on the expression in serum in multiple patient conditions, we referred to the Cancer Proteomic Database (Arntzen et al., 2015) for proper blood expression pattern under specific pathogenic conditions, which is also correspondent with our prediction.

The third rule derived from dataset-2 also involves two parameters ELIAFRDFNWRGGVVAGGSC and KWKQDYINNHFVKVNRTGSC with their respective expression tendencies in patient samples. Based on BLAST, such two peptides have be accurately realigned to two specific proteins, namely, transmembrane protein 39B (Altschul et al., 1990; Mount, 2007; Kim et al., 2013; Pruitt et al., 2014) and fructosamine-3-kinase, contributing to the identification of glioblastoma multiforme patients. According to such rules, both identified proteins are upregulated during tumorigenesis. Recent publications (Delplanque et al., 2004; Kim et al., 2013; Nass et al., 2014) have validated that the specific expression patterns of such two proteins during the initiation and progression of glioblastoma multiforme turn out to be up-regulation, corresponding with our prediction rules. Due to the lack of serum-based proteomic studies for multiple diseases subtypes, the unique expression patterns of such two genes in blood/serum have been partially verified by referring to the data released from the Cancer Proteomic Database (Arntzen et al., 2015).

All the identified genes in this work are the source of identified immunogenic antigens and are functionally related to tumorigenesis. All the quantitative rules have been validated by recent proteomic analysis, confirming the efficacy and accuracy of our prediction. Therefore, our study settles up a systematic computational workflow for the identification of potential immunosignature in multiple cancer subtypes at the proteomics level, providing new insights into the immunogenic characteristics of tumorigenesis.

## Data Availability Statement

Publicly available datasets were analyzed in this study. This data can be found here: https://www.ncbi.nlm.nih.gov/geo/query/acc.cgi?acc=GSE52582.

## Author Contributions

TH and Y-DC designed the study. XP and LC performed the experiments. TZ, Y-HZ, and YZ analyzed the results. LC, XP, and TZ wrote the manuscript. All authors contributed to the research and reviewed the manuscript.

### Conflict of Interest

The authors declare that the research was conducted in the absence of any commercial or financial relationships that could be construed as a potential conflict of interest.
